# Functional dosimetric metrics for predicting radiation-induced lung injury in non-small cell lung cancer patients treated with chemoradiotherapy

**DOI:** 10.1186/1748-717X-7-69

**Published:** 2012-05-17

**Authors:** Dongqing Wang, Jinbo Sun, Jingyu Zhu, Xiaohong Li, Yanbo Zhen, Songtao Sui

**Affiliations:** 1 Department of Radiation Oncology, Shandong Cancer Hospital, Shandong Academy of Medical Sciences, Jinan, People’s Republic China; 2 Jinan Central Hospital affiliated to Shandong University, Jinan, People’s Republic China; 3 The First People’s Hospital of Qndao Economic and Technical Development Zone, Qingdao, People’s Republic China; 4 Jinan Central Hospital affiliated to Shandong University, 105 Jiefang Road, Jinan, 250012, People’s Republic China

**Keywords:** Radiation therapy, Radiation-induced lung injury, Non-small cell lung cancer, Dose-volume histogram, Single photon emission computed tomography

## Abstract

**Background:**

Radiation-induced lung injury (RILI) is an important dose-limiting toxicity during thoracic radiotherapy. The purpose of this study is to investigate single photon emission computed tomography (SPECT) perfusion-weighted functional dose-volume histogram (FDVH) for predicting RILI in non-small cell lung cancer (NSCLC) patients treated with definitive chemoradiotherapy.

**Methods:**

Fifty-seven locally advanced NSCLC patients receiving chemoradiotherapy were enrolled prospectively. Patients had treatment scans and dose calculations to provide a standard dose-volume histogram (DVH). Fusion of SPECT and computed tomography scans provided perfusion-weighted FDVH and associated functional dosimetric parameters (relative volumes of functional lung receiving more than a threshold dose of 5 – 60 Gy at increments of 5 Gy [FV5 – FV60]). The predictive abilities of FDVH and DVH were calculated and compared based on the area under receiver operating characteristic (ROC) curve (AUC).

**Results:**

The accumulative incidence of ≥ 2 grade RILI was 19.3% with a median follow-up of 12 months. Univariate analysis showed that the functional (FV5 – FV60) and standard (V5 – V40) parameters were associated with RILI (all value of p < 0.05). Close correlations between a variety of functional and standard parameters were found. By ROC curve analysis, functional metrics (AUCs were 0.784 – 0.869) provided similarly (p value 0.233 – 1.000) predictive outcome to standard metrics (AUCs were 0.695 – 0.902) in lower – median dose level parameters (FV5 – FV40). However, FDVH seemed to add some predictive value in higher dose level, the best statistical significance for comparing FV60 with V60 was 0.693 vs. 0.511 (p = 0.055).

**Conclusions:**

Functional metrics are identified as reliable predictors for RILI, however, this observation still needs to be further verified using a larger sample size.

## Background

Platinum-based chemoradiotherapy represents the current treatment standard for locally advanced non-small cell lung cancer (NSCLC). However, treatment success is constrained by poor local control and radiation-induced lung injury (RILI). According to recent data [1-4], clinical symptom RILI (Grade ≥ 2) has been reported to occur in 7.0 – 32.0%, severe RILI (Grade ≥ 3) 2.6 – 18.0%, and the lethal RILI (Grade 5) 0 – 2.0%, for patients receiving definitive chemoradiotherapy.

Recently, multiple risk factors associated with the development of RILI have been identified in the literatures, such as dosimetric factors [1,5-20] (typically the mean lung dose and relative volume of lung receiving more than a threshold dose), biomarkers [21,22] (interleukin-6, transforming growth factor-beta, et cetera), single nucleotide polymorphisms (SNPs) [23-25], and clinical factors [1,5,6,9,12,14,16,17]. However, for each individual patient, there are presently no golden standardized factors for predicting RILI following radiation therapy (RT).

In clinical practice, the dose-volume histogram (DVH) parameters, such as mean lung dose (MLD) and V20, are the most commonly used predictors for RILI. However, these parameters are not ideal due to their limited predictive ability [26], which is probably because of the potential interpatient difference related to inherent radiation sensitivity and base-line pulmonary function are not considered when constructing DVH parameters. Lind et al. [6] and Nioutsikou et al. [27] considered functional parameters, that is standard dosimetric factors plus the pre-RT pulmonary functional information, could improve the predictive outcome. Previous studies [28-31] from Netherlands and Duke University have confirmed regional lung damage, assessed by single photon emission computed tomography (SPECT) perfusion combining the three dimensional dose distribution, was predictive for the overall pulmonary function changes and possibly for the prediction of RILI. However, this functional metrics did not add further predictive value as anticipation, and failed to identify patients group at relatively high or low risks of RILI prospectively [14].

In order to better identify the functional metrics in prediction RILI following chemoradiotherapy in NSCLC patients, present study prospectively recruited a moderate homogenous patient population to further examine the predictive value of functional metrics.

## Methods

### Eligibility and patient population

Fifty-seven locally advanced, unresectable NSCLC patients enrolled in a prospective phase II study from March 2006 to April 2010 were analyzed. Eligibility criteria included biopsy-proven NSCLC with clinical stage IIIA and IIIB, no prior chemotherapy or radiotherapy, no concurrent malignancy and no past history of lung cancer, Karnofsky Performance Status (KPS) scale ≥ 80, life expectancy > 6 months, patients without severe complications, such as chronic obstructive pulmonary disease (baseline of forced expiratory volume in 1.0 second < 40% predicted). Of recruited patients, sixteen (28.1%) were recorded in stage IIIA (N2), this cohort of patients treated with definitive chemoradiotherapy were considered surgically unresectable. To minimize potential confounding factors, only those patients receiving definitive chemotherapy and three dimensional radiotherapy were included. Selective lymph nodes irradiation was never adopted. The protocol was approved by our institutional review board, and written informed consent was obtained from patients.

As part of this study, patients had pre-RT assessments of base-line lung function including symptom assessment, pulmonary function tests (PFTs), and SPECT (Infinia; GE) lung perfusion imaging. A pretreatment positron emission tomography/computed tomograpy (PET/CT; 4 slice Discovery LS; GE) scan was obtained for cancer staging and treatment planning.

### Treatment planning and delivery

Patients were immobilized and underwent dedicated 18F-fluorodeoxyglucose (18F-FDG) PET/CT scanning in the treatment position. A SPECT scan was acquired after planning PET/CT acquisition and before RT. With the 99m-technetium (99m-Tc)-labeled macroaggregated albumin (MAA) was injected intravenously. The same immobilization device was used in both the SPECT scan and planning PET/CT scan. The reconstruction and coregistration of images were performed as previously described [28-31]. The 18F-FDG PET/CT image was used to delineate the gross tumor volume (GTV) following the International Commission on Radiological Units recommendations, including the primary disease plus any involved regional lymph nodes as determined by size on the CT scan to be ≥ 1 cm or FDG-avid lymph nodes, regardless of their anatomic size. Before commencing the visual contouring, a diagnostically adequate window for image display was adjusted with the assistance of our nuclear medicine physician. The planning target volume (PTV) was considered to include the GTV plus a 10- to 15-mm margin. Ninety-five percent isodose line encompassed the PTV. Normal tissues (esophagus, spinal cord, heart, and normal lung) were contoured as usual. In particular, functional lung (FL) was weighted by 99m-Tc-MAA SPECT lung perfusion with a threshold of 30% of the maximum radioactivity [29]. It is assumed that perfusion is proportional to function [31]. We delineated the regional well-perfused lung contours as FL.

Based on these functional information, PET/CT/SPECT-guided radiotherapy planning was optimized using Philips Pinnacle^3^ planning system (Philips Radiation Oncology Systems, Milpitas, CA). Generally, we preferred to three dimensional conformal radiotherapy (3D CRT), however, because of complicated target the intensity-modulated radiotherapy (IMRT) technique was also performed some times. For 3D CRT, four or five beams were consistently employed in the treatment plans, typically anterior-posterior beams in combination with oblique beams. In IMRT plans, five to seven beam angles were usually employed for dose optimization. During the optimization, beem angles were guided by perfusion image in order to reduce dose distribution in FL as soon as possible. Dose calculations were performed using Pinnacle^3^ version 7.6c (ADAC, Milpitas, CA) with tissue heterogeneity correction. Planning objective for total lung V20 limited to 37%. The treatment plans were reviewed by peers and delivered using 6 MV beams on linear accelerators. All plans adopted with late-course accelerated hyperfractionated radiotherapy (LCAHRT): the first phase was implemented with the conventional fractionated irradiation. This PTV was defined as receiving 40.0 Gy in total, 2.0 Gy per fraction, five fractions a week. In the second phase, accelerated hyperfractionated radiation was employed. The dose was delivered at 1.4 Gy per fraction, twice daily with a minimum interval of 6 hours, 10 fractions a week to 19.6 – 28.0 Gy in 14 – 20 fractions. The total dose delivered of the two-phase irradiation would be 59.6 – 68.0 Gy/34 – 40 fractions in 5.4 – 6.0 weeks. All patients received 2 – 4 cycles of concurrent or sequential chemotherapy with cisplatin-based (25.0 mg/m^2^ × 3 days) regimens. The chemotherapy regimens used in this study were known to possess similar toxicity and effectiveness for treatment of locally advanced NSCLC [32].

### Standard dose-volume histogram

The DVH was calculated based on the absolute total dose without adjustments for fraction size or overall treatment time. Normal lung was defined as the total lung excluding GTV, trachea, and main bronchi. DVH parameters for normal lung were computed from the 3D dose distributions and were exported from treatment plans. The percentage of lung volume that received more than a threshold dose of radiation were calculated, where the values of threshold dose ranged from 5 to 60 Gy at increments of 5 Gy (V5 – V60).

### Functional dose-volume histogram

The 3D SPECT data were transferred electronically from Nuclear Medicine Center to Radiation Oncology via an internal network. Software in PLUNC (X Fusion) was used to visually superimpose the SPECT images with pre-RT lung contours [14]. After a SPECT scan was adequately registered with the CT data set, the SPECT image was resampled by tri-linear interpolation to match the spatial sampling of the CT data set. The entire 3D RT dose distributions were overlaid on to the SPECT scan. The percentage of SPECT counts in each dose bin was used to generate a “dose SPECT-count histogram”. As it is assumed that perfusion is proportional to function [31], this histogram is termed a functional dose-volume histogram (FDVH) [6,14,27,30]. From the FDVH, the percent of FL receiving from 5 to 60 Gy at increments of 5 Gy were obtained (FV5 – FV60). The adjustment of the dose to the biologically equivalent dose by conventional fractionation size at 2 Gy was not carried out.

### Follow-up and RILI evaluation

The clinical evaluation of patients was performed weekly during the course of RT. Follow-up examinations were performed at 1, 3, 6, 9,12 months, and then 6-month intervals after completion of RT. Pre-RT assessments of lung function include symptom assessment, PFTs, SPECT lung perfusion, as well as a whole body PET/CT scan. Spiral CT-scans of the chest were performed at the end of treatment, and at every follow-up examination to monitor morphological changes in lung structure with respect to RILI. In our analysis, the RILI grade was defined according to the National Cancer Institute Common Toxicity Criteria, version 3.0. [33]. The development of RILI was considered as a binary variable: “no-RILI” (Grade ≤ 1) and “RILI” (Grade ≥ 2).

### Statistical analysis

Differences of functional and standard dosimetric parameters between patients with and without RILI were compared by independent sample t test. Univariate (Chi-square) analysis was used to evaluate the impact of clinical factors, functional and standard dosimetric parameters on the development of RILI. The relationship between functional and standard factors was testing with Pearson correlation r. Receiver operating characteristic (ROC) curves were used to identify the reference threshold of potential predictors and to assess their predictability of the parameters. A higher area under the ROC curve (AUC) indicates a more powerful predictor. The AUCs for functional and standard dosimetric factors were used to statistically test for difference between them. All statistical tests were two-tailed and were performed using statistical software programs SPSS V.16.0. A p value of <0.05 was considered significant.

## Results

### Patient, tumor, and treatment characteristics

The characteristics of patients were summarized in Table 1. The median age was 68 years (range 34 – 71 years). Of the 57 patients, 48 (84.2%) were male and 9 (15.8%) were female; 19 (33.3%) had clinical stage IIIA and 38 (66.7%) had stage IIIB. Seventy-nine percent of patients had a history of smoking. The median baseline forced expiratory volume in 1.0 sec was 2.08 liters (range 0.62 – 3.59 liters).

**Table 1:** Patient, tumor, and treatment characteristics, and association with radiation-induced lung injury

**Characteristic**	**No. of patients (%)**	**p value**
Gender		0.380
Male: Female	48 (84.2): 9 (15.8)	
Age (y)		0.750
≥ 60: < 60	36 (63.2): 21 (36.8)	
Karnofsky Performance Status		0.470
80: >80	13 (22.8): 44 (77.2)	
Histopathology		-
Squamous cell carcinoma	28 (49.1)	
Adenocarcinoma	20 (35.1)	
Large cell carcinoma	3 (5.3)	
NSCLC, not otherwise specified	6 (10.5)	
Clinical stage		1.000
IIIA: IIIB	19 (33.3): 38 (66.7)	
Smoking history		0.702
No: Yes	12 (21.1): 45 (78.9)	
Chemotherapy		0.301
Concurrent: Sequential	17 (29.8): 40 (70.2)	
Primary tumor location		0.087
Upper: Middle and lower lobe	25 (43.9): 32 (56.1)	

For this limited patient population, there was no significant difference in the distribution of clinical parameters (gender, age, KPS, smoking history) between the two groups of patients with and without RILI. Moreover, no tumor-related (tumor location, clinical stage) or treatment-related factor (chemotherapy) was found difference between “RILI” and “no-RILI” groups (all value of p > 0.05).

### Treatment toxicity

Of the 57 patients analyzed, 46 (80.7%) developed grade 0 – 1 RILI, eighty percent of whom were asymptomatic but presented in focal or minimal fibrosis on chest CT images during the follow-up. Seven (12.3%) patients experienced grade 2 RILI, and 3 (5.3%) grade 3. One female patient died of grade 5 RILI after treatment. The accumulative incidence of grade 2 or worse RILI was 19.3% with a median follow-up of 12 months. In patients with RILI, RILI was accompanied by worsening of respiratory symptoms with deterioration of lung function parameters and radiological changes in chest CT-scans.

### Dose-volumetric parameters

**Figure 1:**
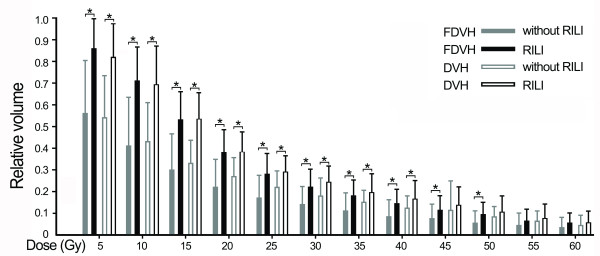
**Comparison for mean value of functional and standard dosimetric parameters between patients with and without radiation-induced lung injury (RILI).** Error bar represents with 1SD, asterisk represents with statistically significant difference.

**Figure 2:**
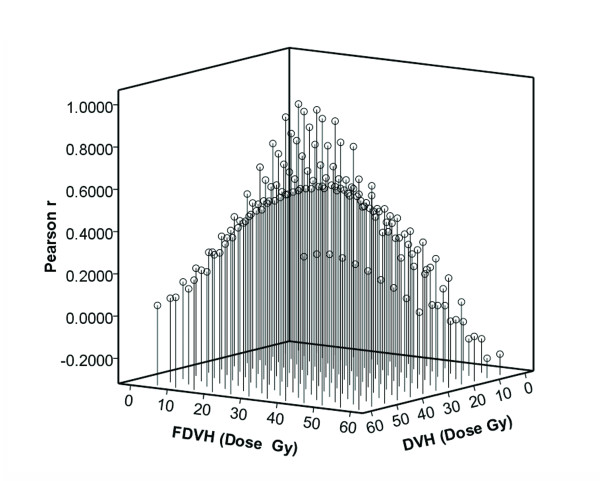
**Pearson correlation coefficient between functional and standard dosimetric parameters**.

**Figure 3:**
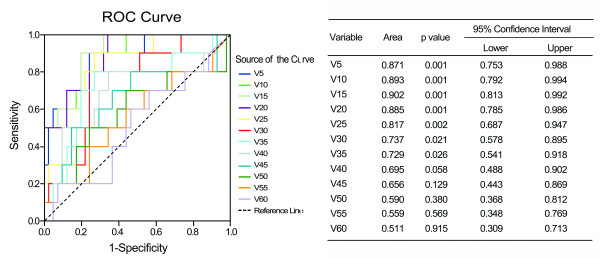
**ROC curve analysis for standard metrics as predictors for radiation-induced lung injury**.

**Figure 4:**
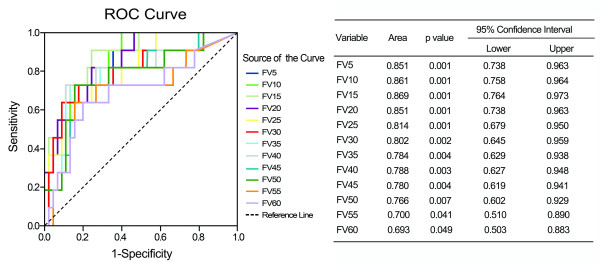
**ROC curve analysis for functional metrics as predictors for radiation-induced lung injury**.

There were significant differences of functional parameters FV5 – FV50 at increments of 5 Gy between patients with and without RILI (p value 0.001 – 0.041), similar results were also found in standard parameters from V5 to V40 (p value 0.001 – 0.040) (Figure 1). By univariate analysis, we found a variety of FDVH (FV5 – FV60) and standard DVH (V5 – V40) parameters were statistically significant (p < 0.05) association with RILI, and the numerical findings were displayed in Table 2. Because of colinearity between FDVH and DVH parameters (Figure 2), we did not perform multivariate analysis. However, we further tested the predictability for these potential predictors using ROC curve. Figure 3 demonstrated the standard DVH as predictors for RILI. Combining with numerical findings, we confirmed V5 – V35 were significantly predictive for RILI (p < 0.05), AUCs ranged from 0.729 (95% confidence interval [CI] 0.541 – 0.918, p = 0.026) to 0.902 (95% CI 0.813 – 0.992, p = 0.001), furthermore, V40 was observed a borderline significant predictor with a predictive ability of 0.695 (95% CI 0.488 – 0.902, p = 0.058). However, V45 – V60 failed to identify patients who were at risk of developing RILI (AUCs were 0.511 – 0.656, p > 0.1). Comparing with standard DVH parameters, functional parameters provided equally or slightly better predictive outcome. As indicated in Figure 4, all the ROC curves of functional parameters were aboved the predictive reference line. Functional parameters observed in present study (FV5 – FV60) could successfully distinguish patients at risk for RILI from current cohort of patients. In order to better ascertain their prognostic value to predict RILI comparing with standard DVH parameters, we compared predictability based on the area beneath ROC curve. For parameters in lower and median dose level (5 – 40 Gy), the FDVH yielded similar predictability to standard DVH, the difference of AUCs was less than 0.1 (p value 0.233 – 1.000). As for parameters in higher dose level (40 – 60 Gy), the functional metrics generally provided better predictability to their counterparts, the difference between ROC areas even reached to 0.2 at 60 Gy dose level (0.693 vs. 0.511, p = 0.055) (Table 3).

**Table 2:** Univariate analysis of functional and standard dosimetric parameters association with radiation-induced lung injury (RILI)

**Parameters**	^**a**^**Cut-off value**	**RILI rate (%)**	**p value**
**FV5** / V5	**0.80** / 0.70	^**b**^**5.3:**^**c**^**47.4***/*^b^5.4: ^c^45.0	**0.001** / 0.001
**FV10** / V10	**0.45** / 0.56	**5.4: 45.0** / 5.1: 50.0	**0.001** / 0.001
**FV15** / V15	**0.40** / 0.40	**2.9: 43.5** / 0.0: 39.3	**0.001** / 0.001
**FV20** / V20	**0.30** / 0.32	**5.6: 42.9** / 3.2: 38.5	**0.001** / 0.001
**FV25** / V25	**0.28** / 0.26	**9.1: 53.8** / 5.9: 39.1	**0.001** / 0.004
**FV30** / V30	**0.21** / 0.21	**7.7: 44.4** / 8.6: 36.4	**0.002** / 0.015
**FV35** / V35	**0.15** / 0.17	**7.3: 50.0** / 7.9: 42.1	**0.001** / 0.004
**FV40** / V40	**0.14** / 0.17	**7.1: 53.3** / 7.1: 31.0	**0.001** / 0.041
**FV45** / V45	**0.11** / 0.13	**7.1: 53.3** / 9.7: 30.8	**0.001** / 0.089
**FV50** / V50	**0.07** / 0.11	**7.1: 53.3** / 12.8: 37.5	**0.001** / 0.061
**FV55** / V55	**0.06** / 0.08	**9.5: 50.0** / 15.8: 31.3	**0.003** / 0.270
**FV60** / V60	**0.04** / 0.03	**10.0: 43.8** / 15.0:22.6	**0.008** / 0.721

Note: functional dosimetric parameters associated with RILI were typed with bold.

a Cut-off value was optimized by receiver operating characteristic curve.

b RILI rate calculated when Vx < cut-off value.

c RILI rate calculated when Vx ≥ cut-off value.

**Table 3:** Pairwise comparison of AUCs between functional and standard dosimetric parameters for predicting radiation-induced lung injury

**Parameters**	^**a**^**DBA**	**p value**	**95% Confidence Interval**	
			**Lower**	**Upper**
FV5 vs. V5	0.020	0.922	−0.113	0.125
FV10 vs. V10	0.032	0.819	−0.075	0.095
FV15 vs. V15	0.033	0.770	−0.090	0.122
FV20 vs. V20	0.034	1.000	−0.146	0.146
FV25 vs. V25	0.003	0.655	−0.127	0.202
FV30 vs. V30	0.065	0.308	−0.082	0.260
FV35 vs. V35	0.055	0.564	−0.114	0.209
FV40 vs. V40	0.093	0.233	−0.064	0.261
FV45 vs. V45	0.124	0.114	−0.033	0.308
FV50 vs. V50	0.176	0.081	−0.019	0.323
FV55 vs. V55	0.141	0.120	0.029	0.254
FV60 vs. V60	0.182	0.055	−0.004	0.346

Note: AUC area under the receiver operating characteristic curve (ROC).

^a^ DBA: Difference between ROC areas.

## Discussion

At present, the mechanisms of RILI have not yet been fully understood. Current studies suggest that many factors, including dosimetry [1,5-20], cytokines [21,22], SNPs [23-25], and clinical factors (such as tumor location [5], pre-RT lung function [6,14], age [9], smoking history [16], gender [17], chemotherapy [1,17] and radiotherapy technique [20], et cetera), contribute to the risk of development of RILI, nonetheless, the occurrence of RILI remains unpredictable. Therefore, more reliable predictors or methods in identifying individuals at a high risk of developing RILI are most desirable for early treatment modifications in order to avoid serious complications.

In present study, we developed the SPECT weighted functional dosimetric parameters to attempt to predict lung toxitity induced by LCAHRT. The present report demonstrated that a variety of parameters including standard (V5 – V40) and functional (FV5 – FV60) parameters were significantly associated with RILI, and the results were in accordance with previous studies [8,9,11,12,15-18,22]. For example, in the study by Dang et al. [17], they considered that lung was a parallel organ and so the functional subunits were connected in parallel. Although a large volume of lung with a low dose of radiation would harm the functions of several subunits [11-13,15], the much higher dose of radiation given to a small lung volume might enlarge the impairment progressively and finally lead to the impairment of the whole-lung function [18,19]. It is believed that the factors of dosage and lung volume are equally important to RILI morbidity, which cannot be determined by a single DVH parameter [8,16,17,19]. Due to the colinearity between these parameters confirmed by present (Figure 2 and Additional file 1: Table S1) and prior studies [7,8,12,16,17], we could not induct them into the regression model simultaneously when the multivariate analysis was carried out. As reported in Table 2 of this paper, all the FDVH and most of the DVH parameters were evidently related to the occurrence of RILI. If any one of parameters was above a certain value, there might be a higher risk for development of RILI. Kocak et al. [14] suggested that the precise dosimetric parameter selected was not critical as there was a strong correlation between the different dosimetric parameters as long as the radiation technique being used is relatively uniform across patients. Jin et al. [16] also noted very high correlations among relative volumes of lung exposed to various dose thresholds, therefore they investigated a comprehensive list of dosimetrics parameters rather than an individual dose-volume constraint. The results indicated that if lung DVH met a set of “threshold” constraints, i.e., V20 < 25%, V25 < 20%, V35 < 15%, and V50 < 10%, the incidence of RILI was extremely low, only 2% at 1 year. Until the effects of different dose levels on lung toxicity were better understood, they proposed using the shape of the DVH curve, rather than a single point on DVH, to limit incidence of RILI.

SPECT of 99m-Tc-labeled MAA provides a map of the spatial distribution of lung perfusion, which has been shown to be proportional to lung function. The SPECT image directly correlates the concentration of radiolabeled microspheres to regional blood flow. Perfusion imaging is clinically relevant to lung function because ventilation without perfusion is more common than perfusion without ventilation. This functional information has been used in radiotherapy planning to identify normal functioning lung tissue when treating lung tumors using three-dimensional radiation treatment planning. It is believed that ideal radiation should be delivered in a manner that minimizes its functional consequences. For the most part, this goal has been sought by trying to minimize the volume of computed tomography-defined lung tissue within the treatment fields. This approach does not, however, consider possible variations in the functional competence of different regions of the lung [27,34]. The same problem arises in the interpretation of DVH. From the viewpoint of biophysics, DVH parameters are constructed to consider both lungs as a homogeneous organ. In fact, the vulnerability of the lung to radiation toxicity is presented in the spatial differences [27,34]. Furthermore, co-existent lung diseases in the majority of lung cancer patients result in regional differences in lung function. Therefore, using the FDVH may be more meaningful for plan evaluation and is anticipated to show a better correlation with RILI.

Lind et al. [6] suggested that considered the pre-RT pulmonary function (PFTs and SPECT lung perfusion scans) combining with three-dimensional dose distribution seemed to be best ability to predict outcome. De Jaeger et al. [30] also reported perfusion weighted dosimetric parameters provided a better estimation of lung functional outcome after high-dose radiotherapy of NSCLC than pure dose parameters. Kocak et al. [14] prospectively assessed the dosimetric/functional parameters in two groups patients from Duke and the Netherlands Cancer Institute (NKI) for predicting radiation pneumonitis. In the Duke data, perfusion weighted parameters (FV20 – FV30) had slightly greater AUCs than standard parameters (0.54 – 0.55 vs. 0.51 – 0.54), however, mean perfusion weighted lung dose (MpLD) had slightly lower AUC than MLD (0.59 vs. 0.62). On the contrary, in the NKI data, MpLD appeared as the most significant predictor, better than the nonperfusion weighted parameter (0.71 vs. 0.61).

In present study, perfusion weighted parameters generally provided similarly (AUCs were 0.784 – 0.869 for FDVH vs. 0.695 – 0.902 for DVH) predictive outcome to nonperfusion weighted parameters in lower and median dose level. In respect to parameters in higher dose level, functional metrics appeared to be better than standard metrics (AUCs were 0.693 – 0.788 for FDVH vs. 0.511 – 0.695 for DVH). There was a borderline statistical significance for comparing FV60 with V60 (p = 0.055). As shown in Figure 4, ROC curve distributions for functional parameters in higher dose level were distinctly better than standard parameters displayed in Figure 3, which demonstrated the characteristic of standard parameters as predictors for RILI. From these charts, we found a certain trend that functional metrics improved the predictive outcome in higher dose level. Therefore, considering our limited sample size, we presumed that perfusion weighted functional metrics might be more reliable as predictors for RILI comparing with standard metrics. Moreover, according to report by Nioutsikou et al. [27], functional metrics might be provide better predictive outcome in cases with localized perfusion deficits, while no added benefit in lung tumors with patchy perfusion.

Contemplating the present study, several factors may confine the predictive outcome of using FDVH metrics. Firstly, in our study, sample size is not large enough to reveal such a significant difference. Secondly, we considered a perfusion value of 30% or more of the maximum radioactivity to be functional and the remaining regional lung was not [29]. In fact, normal lung function is similar to a spectrum, the use of a 30% cutoff point creates FL and non-FL, which may result in losing parts of the ‘non-FL’ information or underestimating the function in these regions [28]. Furthermore, the optimized cutoff point for creating FL has not yet been identified, therefore, FDVH parameters derived from the best cutoff point might be more associated with development of RILI. Further studies focusing on FL definition will help to better clarify the characteristic of FDVH metrics for predicting RILI. Thirdly, because of perfusion image was actually used to optimize the radiation plan, this might lead to a reduced sensitivity with regard to the predictive value of functional metrics. At last, the diagnostic uncertainty of radiation pneumonitis may be a factor that makes the prediction of RILI difficult. Pre-existing lung disease, tumor regrowth/progression, and cardiac disease that may confound the diagnosis.

## Conclusions

This prospective study suggests that standard (V5-V40) and functional (FV5-FV60) parameters are potential predictors in the identification of patients at risk of RILI. In general, functional metrics provide similar predictability to standard metrics, and functional parameters in higher dose level seem to be more reliable to their counterparts, however, this observation still needs to be further verified using a larger sample size.

## Abbreviations

RILI, Radiation-induced lung injury; FDVH, Functional dose-volume histogram; AUC, Area under the receiver operating characteristic curve.

## Competing interests

The authors declared that they have no competing interests.

## Authors’ contributions

DW carried out the data collection and manuscript writing; JS helped to conceive the study; JZ made contribution to the design of this study; XL helped to collect data; YZ participated in statistical analysis; SS involved in general supervision of this study. All authors read and approved the final manuscript.

## Supplementary Material

Additional file 1 Appendix Pearson correlation coefficient between functional and standard parameters. Click here for file

## References

[B1] Barriger RB Dose-volume analysis of radiation pneumonitis in non-small-cell lung cancer patients treated with concurrent cisplatinum and etoposide with or without consolidation docetaxel Int J Radiat Oncol Biol Phys 2010 78 1381 1386 10.1016/j.ijrobp.2009.09.030 20231061

[B2] Werner-Wasik M Paulus R Curran WJ Byhardt R Acute esophagitis and late lung toxicity in concurrent chemoradiotherapy trials in patients with locally advanced non-small-cell lung cancer: analysis of the radiation therapy oncology group (RTOG) database Clin Lung Cance 2011 12 245 251 10.1016/j.cllc.2011.03.026 21726824

[B3] Phernambucq EC Outcomes of concurrent chemoradiotherapy in patients with stage III non-small-cell lung cancer and significant comorbidity Ann Oncol 2011 22 132 138 10.1093/annonc/mdq316 20595452

[B4] Saitoh JI Concurrent Chemoradiotherapy Followed by Consolidation Chemotherapy with Bi-Weekly Docetaxel and Carboplatin for Stage III Unresectable, Non-Small-Cell Lung Cancer: Clinical Application of a Protocol Used in a Previous Phase II Study Int J Radiat Oncol Biol Physin press 10.1016/j.ijrobp.2011.03.007 21601375

[B5] Graham MV Clinical dose-volume histogram analysis for pneumonitis after 3D treatment for non-small cell lung cancer (NSCLC) Int J Radiat Oncol Biol Phys 1999 45 323 329 10487552 10.1016/s0360-3016(99)00183-2

[B6] Lind PA Receiver operating characteristic curves to assess predictors of radiation-induced symptomatic lung injury Int J Radiat Oncol Biol Phys 2002 54 340 347 12243806 10.1016/s0360-3016(02)02932-2

[B7] Jenkins P D’Amico K Benstead K Elyan S Radiation pneumonitis following treatment of non-small-cell lung cancer with continuous hyperfractionated accelerated radiotherapy (CHART) Int J Radiat Oncol Biol Phys 2003 56 360 366 10.1016/S0360-3016(02)04491-7 12738310

[B8] Willner J Jost A Baier K Flentje M A little to a lot or a lot to a little? An analysis of pneumonitis risk from dose-volume histogram parameters of the lung in patients with lung cancer treated with 3-D conformal radiotherapy Strahlenther Onkol 2003 179 548 556 10.1007/s00066-003-1078-0 14509954

[B9] Claude L A prospective study on radiation pneumonitis following conformal radiation therapy in non-small-cell lung cancer: clinical and dosimetric factors analysis Radiother Oncol 2004 71 175 181 10.1016/j.radonc.2004.02.005 15110451

[B10] Fay M Dose-volume histogram analysis as predictor of radiation pneumonitis in primary lung cancer patients treated with radiotherapy Int J Radiat Oncol Biol Phys 2005 61 1355 1363 10.1016/j.ijrobp.2004.08.025 15817337

[B11] Yorke ED Correlation of dosimetric factors and radiation pneumonitis for non-small-cell lung cancer patients in a recently completed dose escalation study Int J Radiat Oncol Biol Phys 2005 63 672 682 10.1016/j.ijrobp.2005.03.026 15939548

[B12] Wang S Analysis of clinical and dosimetric factors associated with treatment-related pneumonitis (TRP) in patients with non-small-cell lung cancer (NSCLC) treated with concurrent chemotherapy and three-dimensional conformal radiotherapy (3D-CRT) Int J Radiat Oncol Biol Phys 2006 66 1399 1407 10.1016/j.ijrobp.2006.07.1337 16997503

[B13] Allen AM Fatal pneumonitis associated with intensity- modulated radiation therapy for mesothelioma Int J Radiat Oncol Biol Phys 2006 65 640 645 10.1016/j.ijrobp.2006.03.012 16751058

[B14] Kocak Z Prospective assessment of dosimetric/physiologic-based models for predicting radiation pneumonitis Int J Radiat Oncol Biol Phys 2007 67 178 186 10.1016/j.ijrobp.2006.09.031 17189069 PMC1829491

[B15] Schallenkamp JM Miller RC Brinkmann DH Foote T Garces YI Incidence of radiation pneumonitis after thoracic irradiation: dose-volume correlates Int J Radiat Oncol Biol Phys 2007 67 410 416 10.1016/j.ijrobp.2006.09.030 17236964

[B16] Jin H Dose-volume thresholds and smoking status for the risk of treatment-related pneumonitis in inoperable non-small cell lung cancer treated with definitive radiotherapy Radiother Oncol 2009 91 427 432 10.1016/j.radonc.2008.09.009 18937989 PMC5555233

[B17] Dang J Analysis of related factors associated with radiation pneumonitis in patients with locally advanced non-small-cell lung cancer treated with three- dimensional conformal radiotherapy J Cancer Res Clin Oncol 2010 136 1169 1178 10.1007/s00432-010-0764-4 20130912 PMC11827824

[B18] Roeder F Correlation of patient-related factors and dose-volume histogram parameters with the onset of radiation pneumonitis in patients with small cell lung cancer Strahlenther Onkol 2010 186 149 156 10.1007/s00066-010-2018-4 20165822

[B19] Ramella S Adding ipsilateral V20 and V30 to conventional dosimetric constraints predicts radiation pneumonitis in stage IIIA-B NSCLC treated with combined-modality therapy Int J Radiat Oncol Biol Phys 2010 76 110 115 10.1016/j.ijrobp.2009.01.036 19619955

[B20] Barriger RB A dose-volume analysis of radiation pneumonitis in non-small cell lung cancer patients treated with stereotactic body radiation therapy Int J Radiat Oncol Biol Physin press 10.1016/j.ijrobp.2010.08.056 21035956

[B21] Kim JY The TGF-beta1 dynamics during radiation therapy and its correlation to symptomatic radiation pneumonitis in lung cancer patients Radiat Oncol 2009 4 59 10.1186/1748-717X-4-59 19943923 PMC2789091

[B22] Chen Y Interleukin (IL)-1A and IL-6: applications to the predictive diagnostic testing of radiation pneumonitis Int J Radiat Oncol Biol Phys 2005 62 260 266 10.1016/j.ijrobp.2005.01.041 15850931

[B23] Yuan X Single nucleotide polymorphism at rs1982073:T869C of the TGFbeta 1 gene is associated with the risk of radiation pneumonitis in patients with non-small-cell lung cancer treated with definitive radiotherapy J Clin Oncol 2009 27 3370 3378 10.1200/JCO.2008.20.6763 19380441 PMC4385796

[B24] Hildebrandt MA Genetic variants in inflammation-related genes are associated with radiation-induced toxicity following treatment for non-small cell lung cancer PLoS One 2010 5 e12402 10.1371/journal.pone.0012402 20811626 PMC2928273

[B25] Yang M Association of P53 and ATM polymorphisms with risk of radiation-induced pneumonitis in lung cancer patients treated with radiotherapy Int J Radiat Oncol Biol Phys 2011 79 1402 1407 10.1016/j.ijrobp.2009.12.042 20729006

[B26] Rodrigues G Lock M D’Souza D Yu E Van Dyk J Prediction of radiation pneumonitis by dose-volume histogram parameters in lung cancer – a systematic review Radiother Oncol 2004 71 127 138 10.1016/j.radonc.2004.02.015 15110445

[B27] Nioutsikou E Partridge M Bedford JL Webb S Prediction of radiation-induced normal tissue complications in radiotherapy using functional image data Phys Med Biol 2005 50 1035 1046 10.1088/0031-9155/50/6/001 15798307

[B28] Marks LB Physical and biological predictors of changes in whole-lung function following thoracic irradiation Int J Radiat Oncol Biol Phys 1997 39 563 570 10.1016/S0360-3016(97)00343-X 9336133

[B29] Seppenwoolde Y Radiation dose-effect relations and local recovery in perfusion for patients with non-small-cell lung cancer Int J Radiat Oncol Biol Phys 2000 47 681 690 10.1016/S0360-3016(00)00454-5 10837952

[B30] De Jaeger K Pulmonary function following high-dose radiotherapy of non-small-cell lung cancer Int J Radiat Oncol Biol Phys 2003 55 1331 1340 10.1016/S0360-3016(02)04389-4 12654445

[B31] Boersma LJ A new method to determine dose-effect relations for local lung-function changes using correlated SPECT and CT data Radiother Oncol 1993 29 110 116 10.1016/0167-8140(93)90235-Z 8310136

[B32] Schiller JH Comparison of four chemotherapy regimens for advanced non-small-cell lung cancer N Engl J Med 2002 346 92 98 10.1056/NEJMoa011954 11784875

[B33] Trotti A CTCAE v3.0: development of a comprehensive grading system for the adverse effects of cancer treatment Semin Radiat Oncol 2003 13 176 181 10.1016/S1053-4296(03)00031-6 12903007

[B34] Seppenwoolde Y De Jaeger K Boersma LJ Belderbos JS Lebesque JV Regional differences in lung radiosensitivity after radiotherapy for non-small-cell lung cancer Int J Radiat Oncol Biol Phys 2004 60 748 758 10.1016/j.ijrobp.2004.04.037 15465191

