# Supplementary material for: Functional dosimetric metrics for predicting radiation-induced lung injury in non-small cell lung cancer patients treated with chemoradiotherapy
Source: Radiat Oncol. 2012 May 17;7:69. doi: 10.1186/1748-717X-7-69 (PMC3434088; doi:10.1186/1748-717X-7-69)
Supplement: Additional file 1 — Appendix Pearson correlation coefficient between functional and standard parameters. [file 1748-717X-7-69-S1.doc]

**Appendix** Pearson correlation coefficient between functional and standard dosimetric parameters (Pearson r)

|  | FV5 | FV10 | FV15 | FV20 | FV25 | FV30 | FV35 | FV40 | FV45 | FV50 | FV55 | FV60 | V5 | V10 | V15 | V20 | V25 | V30 | V35 | V40 | V45 | V50 | V55 | V60 |
| --- | --- | --- | --- | --- | --- | --- | --- | --- | --- | --- | --- | --- | --- | --- | --- | --- | --- | --- | --- | --- | --- | --- | --- | --- |
| FV5 |  | 0.944 | 0.851 | 0.748 | 0.644 | 0.549 | 0.456 | 0.390 | 0.317 | 0.248 | 0.157 | 0.114 | 0.846 | 0.829 | 0.786 | 0.676 | 0.501 | 0.369 | 0.244 | 0.136 | -0.024 | -0.090 | -0.160 | -0.222 |
| FV10 | 0.944 |  | 0.950 | 0.866 | 0.767 | 0.669 | 0.571 | 0.495 | 0.413 | 0.329 | 0.215 | 0.159 | 0.799 | 0.836 | 0.813 | 0.711 | 0.547 | 0.418 | 0.289 | 0.166 | -0.006 | -0.072 | -0.145 | -0.227 |
| FV15 | 0.851 | 0.950 |  | 0.956 | 0.869 | 0.782 | 0.687 | 0.611 | 0.530 | 0.446 | 0.328 | 0.272 | 0.689 | 0.747 | 0.787 | 0.710 | 0.559 | 0.444 | 0.331 | 0.221 | -0.025 | 0.017 | -0.040 | -0.108 |
| FV20 | 0.748 | 0.866 | 0.956 |  | 0.968 | 0.909 | 0.828 | 0.765 | 0.697 | 0.621 | 0.510 | 0.456 | 0.592 | 0.666 | 0.739 | 0.731 | 0.637 | 0.550 | 0.460 | 0.360 | 0.055 | 0.181 | 0.122 | 0.070 |
| FV25 | 0.644 | 0.767 | 0.869 | 0.968 |  | 0.978 | 0.917 | 0.863 | 0.805 | 0.737 | 0.633 | 0.581 | 0.480 | 0.557 | 0.642 | 0.691 | 0.659 | 0.604 | 0.532 | 0.445 | 0.120 | 0.291 | 0.238 | 0.198 |
| FV30 | 0.549 | 0.669 | 0.782 | 0.909 | 0.978 |  | 0.969 | 0.926 | 0.878 | 0.816 | 0.724 | 0.675 | 0.387 | 0.463 | 0.550 | 0.631 | 0.646 | 0.624 | 0.577 | 0.508 | 0.173 | 0.382 | 0.336 | 0.305 |
| FV35 | 0.456 | 0.571 | 0.687 | 0.828 | 0.917 | 0.969 |  | 0.981 | 0.946 | 0.894 | 0.819 | 0.772 | 0.310 | 0.394 | 0.483 | 0.584 | 0.623 | 0.626 | 0.615 | 0.575 | 0.230 | 0.471 | 0.426 | 0.398 |
| FV40 | 0.390 | 0.495 | 0.611 | 0.765 | 0.863 | 0.926 | 0.981 |  | 0.987 | 0.954 | 0.886 | 0.847 | 0.249 | 0.328 | 0.417 | 0.524 | 0.578 | 0.603 | 0.623 | 0.615 | 0.277 | 0.538 | 0.492 | 0.465 |
| FV45 | 0.317 | 0.413 | 0.530 | 0.697 | 0.805 | 0.878 | 0.946 | 0.987 |  | 0.989 | 0.938 | 0.909 | 0.189 | 0.263 | 0.355 | 0.475 | 0.549 | 0.591 | 0.629 | 0.640 | 0.322 | 0.603 | 0.562 | 0.542 |
| FV50 | 0.248 | 0.329 | 0.446 | 0.621 | 0.737 | 0.816 | 0.894 | 0.954 | 0.989 |  | 0.972 | 0.955 | 0.139 | 0.205 | 0.298 | 0.427 | 0.518 | 0.576 | 0.628 | 0.654 | 0.354 | 0.653 | 0.619 | 0.601 |
| FV55 | 0.157 | 0.215 | 0.328 | 0.510 | 0.633 | 0.724 | 0.819 | 0.886 | 0.938 | 0.972 |  | 0.992 | 0.077 | 0.132 | 0.223 | 0.365 | 0.475 | 0.548 | 0.617 | 0.660 | 0.370 | 0.700 | 0.701 | 0.679 |
| FV60 | 0.114 | 0.159 | 0.272 | 0.456 | 0.581 | 0.675 | 0.772 | 0.847 | 0.909 | 0.955 | 0.992 |  | 0.058 | 0.107 | 0.199 | 0.342 | 0.455 | 0.532 | 0.605 | 0.656 | 0.373 | 0.717 | 0.717 | 0.700 |
| V5 | 0.846 | 0.799 | 0.689 | 0.592 | 0.480 | 0.387 | 0.310 | 0.249 | 0.189 | 0.139 | 0.077 | 0.058 |  | 0.970 | 0.922 | 0.836 | 0.676 | 0.540 | 0.426 | 0.322 | 0.038 | 0.098 | -0.016 | -0.073 |
| V10 | 0.829 | 0.836 | 0.747 | 0.666 | 0.557 | 0.463 | 0.394 | 0.328 | 0.263 | 0.205 | 0.132 | 0.107 | 0.970 |  | 0.968 | 0.891 | 0.737 | 0.601 | 0.482 | 0.370 | 0.093 | 0.132 | 0.014 | -0.052 |
| V15 | 0.786 | 0.813 | 0.787 | 0.739 | 0.642 | 0.550 | 0.483 | 0.417 | 0.355 | 0.298 | 0.223 | 0.199 | 0.922 | 0.968 |  | 0.951 | 0.805 | 0.674 | 0.558 | 0.446 | 0.127 | 0.216 | 0.106 | 0.045 |
| V20 | 0.676 | 0.711 | 0.710 | 0.731 | 0.691 | 0.631 | 0.584 | 0.524 | 0.475 | 0.427 | 0.365 | 0.342 | 0.836 | 0.891 | 0.951 |  | 0.939 | 0.846 | 0.743 | 0.635 | 0.271 | 0.419 | 0.320 | 0.267 |
| V25 | 0.501 | 0.547 | 0.559 | 0.637 | 0.659 | 0.646 | 0.623 | 0.578 | 0.549 | 0.518 | 0.475 | 0.455 | 0.676 | 0.737 | 0.805 | 0.939 |  | 0.973 | 0.902 | 0.810 | 0.411 | 0.625 | 0.541 | 0.492 |
| V30 | 0.369 | 0.418 | 0.444 | 0.550 | 0.604 | 0.624 | 0.626 | 0.603 | 0.591 | 0.576 | 0.548 | 0.532 | 0.540 | 0.601 | 0.674 | 0.846 | 0.973 |  | 0.967 | 0.901 | 0.480 | 0.752 | 0.681 | 0.630 |
| V35 | 0.244 | 0.289 | 0.331 | 0.460 | 0.532 | 0.577 | 0.615 | 0.623 | 0.629 | 0.628 | 0.617 | 0.605 | 0.426 | 0.482 | 0.558 | 0.743 | 0.902 | 0.967 |  | 0.974 | 0.433 | 0.856 | 0.785 | 0.727 |
| V40 | 0.136 | 0.166 | 0.221 | 0.360 | 0.445 | 0.508 | 0.575 | 0.615 | 0.640 | 0.654 | 0.660 | 0.656 | 0.322 | 0.370 | 0.446 | 0.635 | 0.810 | 0.901 | 0.974 |  | 0.441 | 0.938 | 0.867 | 0.811 |
| V45 | -0.024 | -0.006 | -0.025 | 0.055 | 0.120 | 0.173 | 0.230 | 0.277 | 0.322 | 0.354 | 0.370 | 0.373 | 0.038 | 0.093 | 0.127 | 0.271 | 0.411 | 0.480 | 0.433 | 0.441 |  | 0.986 | 0.929 | 0.884 |
| V50 | -0.090 | -0.072 | 0.017 | 0.181 | 0.291 | 0.382 | 0.471 | 0.538 | 0.603 | 0.653 | 0.700 | 0.717 | 0.098 | 0.132 | 0.216 | 0.419 | 0.625 | 0.752 | 0.856 | 0.938 | 0.986 |  | 0.972 | 0.940 |
| V55 | -0.160 | -0.145 | -0.040 | 0.122 | 0.238 | 0.336 | 0.426 | 0.492 | 0.562 | 0.619 | 0.701 | 0.717 | -0.016 | 0.014 | 0.106 | 0.320 | 0.541 | 0.681 | 0.785 | 0.867 | 0.929 | 0.972 |  | 0.984 |
| V60 | -0.222 | -0.227 | -0.108 | 0.070 | 0.198 | 0.305 | 0.398 | 0.465 | 0.542 | 0.601 | 0.679 | 0.700 | -0.073 | -0.052 | 0.045 | 0.267 | 0.492 | 0.630 | 0.727 | 0.811 | 0.884 | 0.940 | 0.984 |  |
